# Artificial Intelligence Approaches for EEG Signal Acquisition and Processing in Lower-Limb Motor Imagery: A Systematic Review

**DOI:** 10.3390/s25165030

**Published:** 2025-08-13

**Authors:** Sonia Rocío Moreno-Castelblanco, Manuel Andrés Vélez-Guerrero, Mauro Callejas-Cuervo

**Affiliations:** Software Research Group, Universidad Pedagógica y Tecnológica de Colombia, Tunja 150002, Colombia; sonia.moreno01@uptc.edu.co (S.R.M.-C.); manuel.velez@uptc.edu.co (M.A.V.-G.)

**Keywords:** artificial intelligence (AI), brain–computer interface (BCI), decomposition algorithms, EEG, lower limbs, motor imagery, multimodal approaches, neurorehabilitation, PRISMA, signal processing

## Abstract

**Highlights:**

**What are the main findings?**

- This review highlights that advanced machine learning algorithms and multimodal fusion strategies have improved the accuracy and robustness of lower-limb motor imagery classification, pointing to a trend toward portable, low-power BCI devices optimized for fewer EEG channels.

**What is the implication of the main finding?**

- These developments pave the way for clinically viable BCIs that are accessible, adaptable, and suitable for real-world neurorehabilitation contexts.

Coordinated efforts are needed to create standardized, open-access datasets and protocols to accelerate translation and adoption into broader contexts.

**Abstract:**

Background: Motor imagery (MI) is defined as the cognitive ability to simulate motor movements while suppressing muscular activity. The electroencephalographic (EEG) signals associated with lower limb MI have become essential in brain–computer interface (BCI) research aimed at assisting individuals with motor disabilities. Objective: This systematic review aims to evaluate methodologies for acquiring and processing EEG signals within brain–computer interface (BCI) applications to accurately identify lower limb MI. Methods: A systematic search in Scopus and IEEE Xplore identified 287 records on EEG-based lower-limb MI using artificial intelligence. Following PRISMA guidelines (non-registered), 35 studies met the inclusion criteria after screening and full-text review. Results: Among the selected studies, 85% applied machine or deep learning classifiers such as SVM, CNN, and LSTM, while 65% incorporated multimodal fusion strategies, and 50% implemented decomposition algorithms. These methods improved classification accuracy, signal interpretability, and real-time application potential. Nonetheless, methodological variability and a lack of standardization persist across studies, posing barriers to clinical implementation. Conclusions: AI-based EEG analysis effectively decodes lower-limb motor imagery. Future efforts should focus on harmonizing methods, standardizing datasets, and developing portable systems to improve neurorehabilitation outcomes. This review provides a foundation for advancing MI-based BCIs.

## 1. Introduction

Motor imagery (MI) is defined as the cognitive ability to internally reproduce motor actions through cortical activation, without actual physical execution and with the inhibition of the corresponding muscular activity [1,2]. This process involves the activation of specific cortical regions and the integration of neural signals that support the prediction, planning, and mental simulation of actions [3,4].

MI processes reflect the brain’s remarkable ability to simulate motor actions through the coordinated activity of multiple regions, including the primary motor cortex, supplementary motor area, basal ganglia, and cerebellum, which are involved in coordination, motor learning, and memory [5,6]. These neural dynamics enable the mental rehearsal and refinement of motor skills without physical execution [7], facilitating both the acquisition and long-term consolidation of motor abilities [8]. This mechanism constitutes a fundamental process for understanding how the brain organizes and optimizes motor functions [9]. Its study through electroencephalographic (EEG) signals, particularly for identifying MI in the lower limbs, represents a key area in the advancement of brain–computer interfaces [10].

Brain–computer interfaces (BCIs) are designed to decode motor intentions and translate them into control commands for external devices such as robotic prostheses and assistive technologies for individuals with disabilities [11]. A key application of EEG-based BCIs involves detecting the neural patterns associated with imagined limb movements. However, this task presents considerable challenges in signal processing and analysis because EEG signals, despite providing high temporal accuracy, exhibit low spatial resolution and low amplitude, making them harder to interpret than signals recorded from other body regions [7].

Despite the increasing number of studies on EEG, BCI, and MI, there is still no clear consensus regarding the most effective methodological approaches. This variability constitutes a critical concern, as differences in experimental design, acquisition protocols, and signal processing techniques can affect the reliability and comparability of results [8]. Some studies focus on the use of multichannel configurations to improve spatial resolution, while others prioritize the simplicity and portability of systems for real-time applications [12,13]. Therefore, the lack of standardization hinders direct comparisons between studies and highlights the need to establish common guidelines.

The application of artificial intelligence (AI) techniques to EEG signal processing has increased, delivering high classification performance and enabling real-time implementation in brain–computer interface systems. However, the diversity of algorithms, preprocessing pipelines, and study designs complicates systematic comparison and clinical translation. To address this gap, the present study conducts a systematic review following the PRISMA (Preferred Reporting Items for Systematic Reviews and Meta-Analyses) [14] guidelines to evaluate the literature on EEG signal acquisition and processing for lower-limb MI using machine learning and deep learning algorithms.

This review surveys current EEG acquisition practices for lower-limb MI; contrasts conventional and AI-based processing pipelines, including emerging portable BCI prototypes; and assesses their degree of methodological standardization. A structured selection of high-quality studies underpins a critical synthesis to steer future research and clinical translation.

The paper is organized into five sections: Section 1 introduces the topic and defines the scope of this review. Section 2 details the methodology, including eligibility criteria, search strategy, and study selection parameters. Section 3 presents the results, highlighting key features of the included studies. Section 4 discusses the findings about the existing literature, and Section 5 concludes with a synthesis of the main insights drawn from the review.

## 2. Methods

This section outlines the methodology applied in the literature review, which was conducted following the PRISMA guidelines [14]. The application of these guidelines ensures a systematic and structured review process based on clearly defined inclusion criteria, thereby enhancing the transparency, reproducibility, and scientific rigor of the study. This approach enables the systematic selection and synthesis of studies within a standardized framework, ensuring objective analysis and facilitating the critical evaluation of their quality, validity, and relevance. Furthermore, by promoting consistency in the reporting of systematic reviews, PRISMA strengthens the reliability of the conclusions drawn from the analyzed evidence [15].

This review was conceived initially as a scoping analysis and was therefore not prospectively registered. Once the scope was refined into a full systematic review, registration was no longer feasible.

### 2.1. Eligibility Criteria

Eligibility criteria included studies that met the following conditions: (i) written in English; (ii) published between January 2020 and April 2025; (iii) focusing on the identification of lower-limb motor imagery; (iv) utilizing electroencephalography (EEG) as the primary neural recording technique; (v) applying artificial intelligence-based methods within a BCI framework for signal processing, classification, or analysis; (vi) addressing applications in neuroscience or motor rehabilitation contexts; (vii) published in the form of experimental studies, clinical research, literature reviews, conference proceedings, symposium contributions, book chapters, or scientific journal articles with full-text availability.

Studies were excluded if they met any of the following criteria: (i) they focused exclusively on upper-limb MI tasks without addressing lower-limb components; (ii) they employed neuroimaging or neurophysiological techniques other than EEG as the primary measurement modality (e.g., fNIRS, MEG, or fMRI); or (iii) they did not incorporate artificial intelligence-based approaches, such as machine learning or deep learning algorithms, in the processing, analysis, or classification of EEG signals.

These exclusion criteria ensured that only studies directly relevant to the analysis of lower-limb motor imagery EEG using machine-learning methods were considered for synthesis.

The review focused on studies published between 2020 and 2025 to capture the advances in EEG acquisition and analysis methods that have improved the precision and clinical applicability of MI detection. While this five-year window reflects the latest methodological developments, it limits the analysis of long-term trends and excludes earlier foundational research. The literature search and study selection were completed in May 2025 using the selected databases.

### 2.2. Search Methodology and Scope

An initial literature search was carried out in the Scopus and IEEE Xplore databases using carefully formulated queries that included multiple keywords related to EEG, signal acquisition and processing, MI, lower limb movements, and BCIs, as outlined in Table 1. These search strategies employed logical operators to ensure a comprehensive and systematic retrieval of relevant studies. Additionally, it is important to note that some articles included in the final review were incorporated during the manuscript revision process, based on peer reviewer feedback and editorial recommendations, extending beyond the results of the initial search.

It is highlighted that no additional filters were applied in the databases. Supplementary searches were conducted through Google Scholar, PubMed, and by manually screening the reference lists of key articles identified during the primary search. These additional sources were not queried using a structured search string; however, all identified records were assessed according to the same predefined inclusion and exclusion criteria.

### 2.3. Study Identification and Screening

Initially, 287 records were retrieved: (*n* = 207) from Scopus and IEEE Xplore, (*n* = 52) from specialized websites, and (*n* = 28) via backward-citation searching. The pre-screening step removed (*n* = 66) duplicates, and (*n* = 12) records were removed for other reasons. Therefore, (*n* = 129) database records remained for title/abstract screening, and (*n* = 14) of these were excluded. The (*n* = 115) remaining database records, together with the (*n* = 80) items captured from websites and citation searches (total = 195), were then sought for full-text retrieval. Of these, 72 reports (28 database-derived and 44 from other sources) could not be obtained, leaving (*n* = 123) reports for detailed eligibility assessment.

A total of 123 full-text records were assessed against 8 predefined exclusion criteria: E1 (no lower-limb motor imagery focus, *n* = 19), E2 (no AI-based processing/classification, *n* = 14), E3 (EEG not primary modality, *n* = 13), E4 (unclear or unreported participant information, *n* = 11), E5 (≤2-page abstract or insufficient methodological detail, *n* = 10), E6 (duplicate dataset, *n* = 9), E7 (non-English full text, *n* = 7), and E8 (retracted or predatory-journal publication, *n* = 5), for a total of 88 exclusions. Following this screening process, 35 studies met all inclusion criteria and were retained for the final synthesis.

This rigorous and structured screening process ensured that only the most relevant and methodologically sound studies were considered, enhancing the validity and reliability of the conclusions drawn from this systematic review. No automation tools were used during screening.

**Figure 1:**
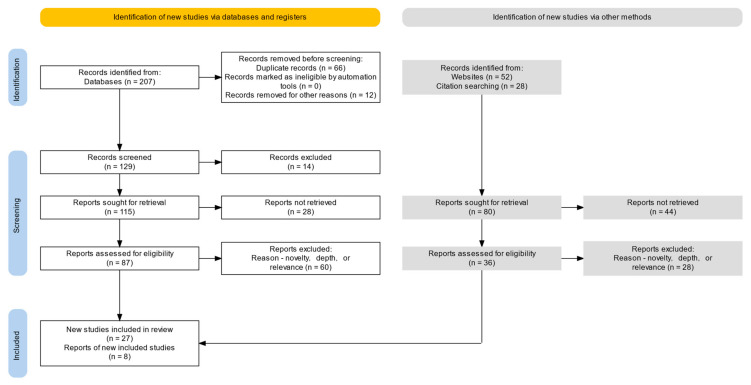
PRISMA flow diagram illustrating the study selection process. The figure details the number of records identified, screened, excluded, and finally included in the systematic review, following PRISMA guidelines.

The study selection process is further detailed in Figure 1, which presents a PRISMA flow diagram outlining each stage of the identification, screening, eligibility assessment, and inclusion of studies [14,15]. This figure provides a visual summary of the methodological steps followed during the systematic review process.

### 2.4. Data Extraction and Items

Data were extracted jointly by the authors using a predefined data extraction form developed in a spreadsheet. For each study, the following items were collected: author(s) and year of publication, study design (experimental, computational, or review), population studied (e.g., healthy subjects, patients, or simulations), EEG acquisition system and configuration, signal preprocessing and filtering methods, feature extraction and selection techniques, classification models and performance metrics, multimodal integration (e.g., EMG or kinematic data), application context (e.g., neurorehabilitation or assistive control), key outcomes and conclusions, and funding sources when reported. No missing data were imputed, and the study authors were not contacted for clarification.

This review did not perform a formal risk of bias assessment for the included studies, as its primary aim was methodological synthesis. Nonetheless, key study limitations, such as small sample sizes, lack of validation, and heterogeneous protocols, were qualitatively noted in the Section 3 and Section 4. Certainty of evidence, including frameworks like GRADE, was not assessed due to the descriptive nature of the review. Given the methodological heterogeneity across studies, a quantitative meta-analysis was not feasible. Instead, the results were synthesized qualitatively by grouping studies into thematic domains including signal acquisition, machine learning data processing, and rehabilitation applications.

## 3. Results

**Figure 2:**
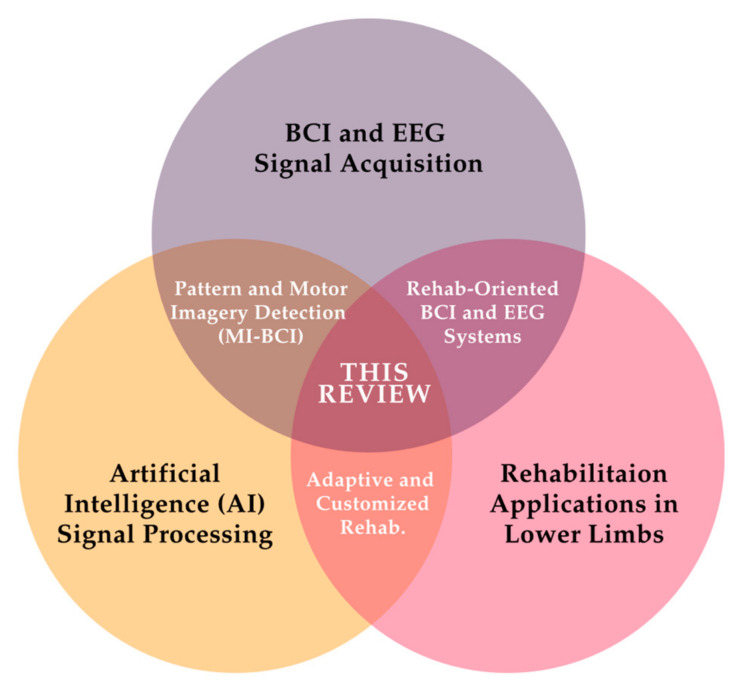
Key thematic domains identified in the systematic review. The figure organizes the included studies into three core categories, providing a structured view of the field.

This section presents the results obtained following the application of the inclusion and exclusion criteria to the selected literature corpus. Figure 2 presents three thematic axes identified through keyword analysis: BCI and EEG signal acquisition, lower-limb rehabilitation applications, and artificial intelligence-based signal processing. These axes correspond directly to the concepts defined in the search strategy and provide the structural scope of the review.

The findings were organized into key domains to allow a structured and comparative interpretation of methodological trends across the selected studies. This categorization reflects each study’s primary research focus while enabling comparison of methodologies, data sources, and system performance. Due to the heterogeneity in study designs and outcome measures, results are presented descriptively. Also, no statistical synthesis or meta-analysis was performed. This approach supports a comprehensive and critical understanding of the contributions.

### 3.1. General Characteristics of the Included Studies

To contextualize the analysis, it is essential first to examine the main features of the studies included in the review. This characterization allows for a better understanding of the methodological diversity and thematic focus present in the selected literature. Table 2 summarizes the key characteristics of the selected studies, including the year of publication, study design, population studied, type of intervention, and the principal findings. This synthesis facilitates the comparison of diverse approaches and outcomes across the body of literature on EEG signal acquisition and processing for MI tasks in the lower limbs.

The acronyms used in Table 2 are provided to standardize and simplify the presentation of information, ensuring clarity and conciseness in the description of the studies reviewed. The column titled Study Design employs the following acronyms: EXP (experimental), COMP (computational), and REV (review). These terms denote the methodological nature of each study, distinguishing between purely experimental works, those integrating computational techniques, computational-only studies, and reviews of existing literature.

Similarly, the Participants column (PCPs) categorizes the populations studied using the following abbreviations: NP (No Participants), HS (Healthy Subjects), PS (Pathological Subjects), and HS + PS (Healthy and Pathological Subjects). This classification differentiates studies that involved no human subjects from those that included healthy volunteers, clinical populations, or a combination of both.

The Sample Size column indicates the number of participants included in the study, when applicable, particularly for experimental papers involving human subjects. The Classes column specifies the number of motor imagery (MI) classes used in the classification process. The Feature Types column refers to the characteristics of the input data provided to the machine learning or signal analysis models. Acronyms used in this column correspond to those defined in the glossary at the end of the document. The Used Techniques column lists the classification models or analytical methods tested or applied, also using acronyms as defined in the glossary. Finally, the Data Type column indicates whether the data used were raw (RAW) or preprocessed (PPD).

Across all these columns, the acronyms NA (Not Available) and NS (Not Specified) may appear, as not all studies provide complete information. Review articles often do not include any of these specific attributes.

Table 2 provides a structured view of the selected studies in this systematic review, highlighting key methodological and demographic aspects. In terms of design, experimental and computational studies predominate, reflecting an interest in evaluating the effectiveness of EEG–BCI interventions for lower limb MI. The use of standardized categories improves the interpretability of the table and highlights trends in methodological approaches, participant demographics, and technological advancements in the field of BCIs.

Regarding participants, there is heterogeneity in the study populations, including healthy individuals and patients with neurological conditions such as spinal cord injury or stroke. Several studies did not involve human participants, relying instead on public datasets or computational simulations. This diversity facilitates comparisons between groups and methodological approaches but also introduces challenges for the generalization and clinical translation of findings.

The described strategies encompass a wide range of approaches, from purely computational analyses and algorithm development to experimental protocols involving MI tasks and the use of assistive technologies such as exoskeletons. Across the surveyed studies, experimental procedures vary in several critical aspects. Differences include electrode montage, duration of MI trials, cue and feedback modalities, preprocessing filters, and classifier training schemes.

Such heterogeneity makes direct comparison difficult and reduces the feasibility of meta-analysis. Absolute uniformity is unrealistic because protocols must adapt to children, adults, and participants with disabilities. Nevertheless, establishing a core set of recommended parameters and reporting standards would improve reproducibility and still permit population-specific adjustments.

Table 2 shows that high decoding accuracy is common once artificial intelligence methods are applied to lower-limb motor imagery EEG. Support Vector Machines (SVMs), Random Forest (RF), convolutional neural networks (CNNs), and long short-term memory (LSTM) networks reach high accuracies. Only about one quarter of the corpus (nine of thirty-five studies) evaluated their pipelines under natural or clinical conditions such as over-ground walking, exoskeleton-assisted gait, or rehabilitation sessions. The remainder used seated laboratory paradigms.

Most workflows begin with feature extraction rather than end-to-end learning. Dominant techniques include spatial filters based on Common Spatial Pattern, multiresolution decompositions such as the Discrete and Wavelet Packet Transforms, and Riemannian geometry descriptors. These features are then provided to the classifier, which is itself part of the artificial intelligence pipeline but not the sole performance driver.

Three recurrent methodological gaps emerge from the reviewed corpus. First, sample sizes are generally small: many studies include fewer than twenty participants, and several rely solely on public datasets or simulations, limiting statistical power and external validity. Second, experimental designs show substantial heterogeneity. Differences in electrode configurations, MI tasks, trial lengths, cue and feedback modalities, preprocessing steps, and classification pipelines hinder meaningful cross-study comparisons. Third, there is a lack of longitudinal evidence. Only a few studies report follow-up assessments beyond the initial sessions or evaluate system performance in valid rehabilitation settings, restricting insights into long-term efficacy and real-world applicability.

Addressing these limitations will require coordinated efforts to conduct multicenter studies with larger and more diverse populations, develop consensus around core experimental protocols, and systematically incorporate extended follow-up measures. While the current evidence demonstrates promising clinical potential for enhanced EEG-based BCI systems, it also underscores the pressing need for more rigorous, standardized, and reproducible research to support their translation into real-world rehabilitation contexts.

### 3.2. Bibliometric Analysis

Understanding the evolution of research on EEG signal acquisition and processing for MI tasks requires a bibliometric perspective that provides valuable insights into how foundational studies influence subsequent works and how research trends emerge and evolve. This section presents a temporal mapping of the reviewed literature, highlighting citation networks and the chronological progression of the studies included in the review.

**Figure 3:**
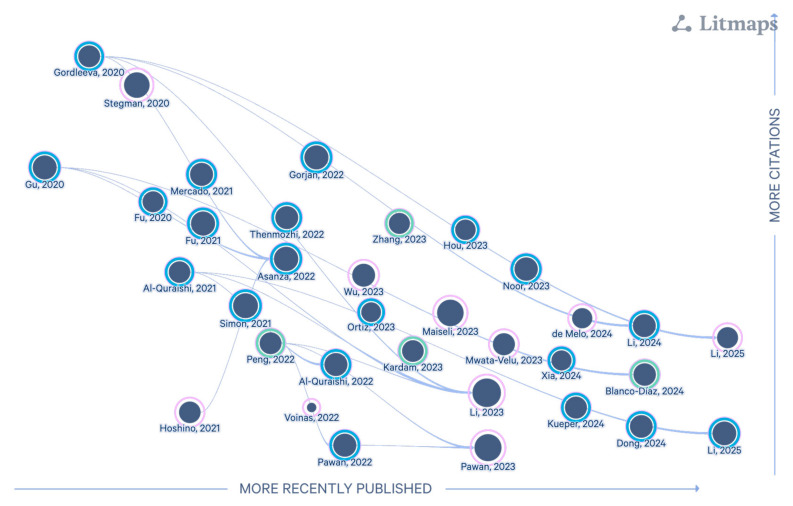
Temporal map of the 35 reviewed articles generated with Litmaps [51]. The graph illustrates the chronological influence between studies, the number of citations received, and the number of references in each article.

Figure 3 below presents a temporal relationship map of the articles included in this review, generated using Litmaps [51]. This visualization highlights the evolution of research over time and reveals key connections between studies, shedding light on conceptual influences, emerging trends, and potential gaps in the existing literature.

Figure 3 provides a visual overview of the temporal and citation-based relationships among the 35 reviewed studies. Each node represents a publication positioned along the x-axis by year (2020–2025). Circle size reflects the number of references cited in each article, serving as a proxy for its informational depth, while ring thickness indicates total citations received as of April 2025 (an approximate measure of scientific impact). Finally, directed arrows show citation paths, with opacity reflecting connection strength.

This visualization highlights how earlier studies have influenced subsequent research, illustrating the evolution of the field and revealing clusters of methodologically related works. The map also helps identify pivotal publications and emerging trends that may inform future directions in AI-based EEG research for motor imagery.

### 3.3. Conceptual Foundations of BCI and EEG Systems

A critical analysis of the literature on motor imagery and EEG-based BCIs requires a clear understanding of the fundamental components and operational mechanisms underlying these systems. BCIs are designed to capture and process brain signals, translating neural activity into control commands for external devices [52]. In the context of neuromotor rehabilitation, BCIs play an essential role by enabling direct communication between cerebral electrical activity and digital control systems, facilitating motor function recovery and patient interaction with assistive technologies [53].

However, despite the rapid technological evolution of BCIs and their growing presence in both scientific research and industrial applications, some of the challenges remain, particularly regarding data privacy and system security. These concerns highlight the need to ensure that advances in system architecture are accompanied by robust mechanisms for the protection of sensitive neural data [54]. Addressing these challenges is critical not only for guaranteeing the security and reliability of BCI applications but also for strengthening their commercial viability and maximizing their societal impact in clinical and real-world contexts [16].

Within this review, clarifying these conceptual foundations is essential to properly contextualize the methodologies, signal processing techniques, and applications discussed in the following sections. This perspective enables a more critical evaluation of current research trends and their implications for the future development of EEG-based BCIs applied to lower-limb motor imagery.

### 3.4. BCI and EEG Signal Acquisition and Processing

**Figure 4:**
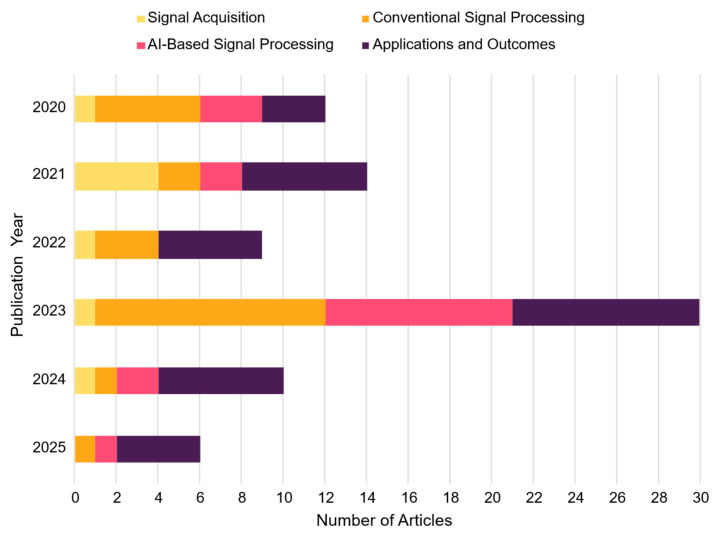
Yearly distribution of methodological focus areas in EEG-based motor imagery studies. Each bar illustrates the relative emphasis on signal acquisition, conventional processing, AI-based processing, and applications across the reviewed literature.

The acquisition of EEG signals constitutes a critical stage in the development of brain–computer interface (BCI) systems, as it directly influences the quality and reliability of the neural data used for subsequent analysis. This section examines the primary methodologies employed in EEG signal acquisition and processing within the context of motor imagery studies. To provide a structured synthesis of the reviewed methodologies, Figure 4 presents a statistical overview of the acquisition parameters, processing strategies, and outcomes identified across the selected studies.

Figure 4 provides a concise snapshot of how the selected literature is distributed across four non-mutually exclusive areas: signal acquisition, conventional signal processing, AI-based signal processing, and applications and outcomes. These are plotted as horizontal stacked bars for each publication year. The total width of a bar represents the cumulative number of theme occurrences in that year, while the length of each segment reflects that theme’s relative contribution. For instance, the 2023 bar is dominated by applications and outcomes along with a substantial AI-based segment, while signal acquisition remains the narrowest slice throughout the timeline. This visualization clarifies prevailing research priorities, revealing a marked shift toward intelligent processing pipelines and practical or clinical deployment in recent years. It also highlights areas where future methodological optimization may be most beneficial.

Since a single study can address several methodological facets, it is counted once for every theme it contributes to. As a result, the segment totals within and across years exceed the 35 unique papers reviewed. Recognizing this overlap is essential for an accurate interpretation of Figure 4 and for the structured discussion that follows. Each study’s multifaceted contributions are assigned to the relevant areas in this and subsequent sections to ensure a comprehensive and nuanced analysis of current practices in EEG-based BCI research for motor imagery applications.

Particular attention is given to the characteristics of the recording systems, including electrode types, electrode placement configurations, sampling rates, and channel selection strategies. The section also examines commonly used filtering and artifact reduction techniques, which are essential for minimizing noise and maintaining neural signal integrity.

In the field of medical engineering, advances in EEG signal acquisition and processing have strengthened the understanding of brain activity. A notable study optimized a movement detection system using EEG signals and motor imagery recognition [17]. The authors addressed the challenge of limited spatial resolution by employing active reference electrodes, which effectively reduce noise. The authors also optimized BCI input processing by identifying optimal electrode pairings tailored to individual users and applied signal transformations to improve result stability. Experiments with 15 participants achieved an average accuracy of 95%, revealing a direct correlation between delta band energy (0–4 Hz) and lower signal variability [17]. These findings offer new insights into brain patterns associated with movement detection.

Despite progress, EEG-based BCI research continues to face challenges in eliminating artifacts and non-neural signals, particularly during motion execution. Several studies have sought to improve EEG signal quality in BCIs, especially in dynamic environments such as locomotion. The contributions of Asanza [18] and Gorjan, et al. [19] are noteworthy for addressing these challenges. Although independent component analysis remains a standard approach for artifact suppression, uncertainty persists regarding the optimal method, depending on movement type and intensity. This highlights the need for well-designed experiments and integrated software–hardware solutions capable of extracting features from adaptive systems for motor imagery task classification.

The acquisition of EEG signals for BCIs and motor imagery detection faces challenges due to their low time–frequency precision, reduced signal-to-noise ratio (SNR), and non-stationary nature. To address these limitations, Kardam, et al. [20] explored decomposition algorithms such as EMD and EEMD. These methods decompose EEG signals into Intrinsic Mode Functions (IMFs), enabling the automatic reconstruction of task-related signals with higher SNR. Ensemble empirical mode decomposition (EEMD) was benchmarked against the conventional empirical mode decomposition (EMD) filtering pipeline, an approach that reconstructs the signal after EMD and then applies the usual narrow-band filtering of the μ and β rhythms. Using only three scalp channels, the EEMD-based preprocessing improved motor-imagery classification accuracy by more than 15% compared with the EMD baseline, demonstrating a clear advantage in signal-to-noise ratio and task discrimination.

Frequency analysis has thus become a widely used tool for studying brain activity during cognitive and motor tasks. For example, Mercado, et al. [21] employed continuous-trajectory reconstruction (CTR) and a multilayer perceptron to infer the flexion and extension torques generated at the hip and knee joints, identifying these joint torques as the kinetic variables decoded from scalp EEG during pre-gait movements. Performance evaluation based on the coefficient of determination, correlation coefficient, and SNR demonstrated higher decoding accuracy for the right lower limb and revealed lateralized electrode patterns specific to each task and joint.

Blanco et al. [22] proposed and compared two ANN-based decoders for estimating lower-limb kinematics during pedaling tasks using delta-band EEG features extracted in 250 ms windows. Recurrent neural networks, particularly LSTM architectures, achieved the best results, with Pearson correlation coefficients close to 0.58 for ankle and knee joint estimations. Performance was validated through kinematic variance analysis, showing accurate estimations during both pedaling and rest phases. Additionally, an inverse relationship was found between pedaling speed and decoder performance, indicating that lower speeds favor more accurate kinematic reconstruction, establishing an important consideration for rehabilitation protocol design.

In recent years, human–machine interfaces (HMIs) for lower-limb rehabilitation systems, particularly exoskeletons, have advanced notably. A major challenge in these applications is the reliable detection of motor intention when integrating multimodal signals such as EEG and electromyography (EMG). To address this, ref. [23] proposed a multimodal approach combining EEG-based motor imagery using event-related (de)synchronization (ERD/ERS) with multichannel EMG signals to improve exoskeleton control. This multimodal approach improves classification accuracy and mitigates typical EEG limitations such as low signal-to-noise ratio and high inter- and intra-subject variability.

Building on this, ref. [24] applied DCA techniques to fuse EEG and EMG features for the recognition of bilateral ankle movements. Using segmentation windows of different sizes, optimal results were obtained with a 250-sample window. Among the classifiers evaluated, LDA achieved the best performance, with recognition accuracy reaching 96.64 ± 4.48%, compared with 89.99 ± 7.94% using EEG alone. Notably, this DCA-based fusion maintained high accuracy even under muscle fatigue conditions, outperforming single-modality systems (*p* < 0.0001).

Noor, et al. [25] developed a predictive model using resting-state EEG to classify patients with moderate traumatic brain injury (TBI) according to their recovery prognosis. Unlike approaches based on complex feature extraction, this study used 60-s segments of raw EEG as direct input to an LSTM neural network. The main contribution lies in simplifying the analysis pipeline through deep learning without extensive preprocessing, improving the model’s clinical applicability. The proposed method achieved a classification accuracy of 87.50 ± 0.05%, outperforming previous studies.

In [26], an iterative source localization method was proposed to select an optimal EEG channel set (OCS: FC1, FC2, C1, C2, Cz), improving performance over the traditional EEG channel set (TCS: C3, C4, Cz). Additionally, a multidomain feature extraction algorithm (MDF) was implemented, combining information from multiple domains. For classification, the study employed an SVM optimized using particle swarm optimization (PSO), achieving an accuracy of 88.43%, representing a 3.35% to 5.41% improvement over traditional methods. This approach enhances the accuracy of MI-BCI systems by reducing irrelevant data and preserving more meaningful EEG information.

Pawan, et al. [27] proposed an optimized method for improving MI-BCI systems, focusing on EEG channel selection, feature extraction, and classification. Using Pearson’s correlation coefficient (PCC), 14 EEG channels from the sensorimotor area were selected, followed by processing using WPD and approximate entropy (ApEn) for feature extraction. Classification with SVM and KNN achieved accuracies of 91.66% and 90.33%, respectively, outperforming previous approaches on the BCI Competition IV dataset [55].

Similarly, ref. [28] emphasized the role of advanced classification and connectivity analysis in motor imagery detection. A cortical source model with 62 regions of interest was defined to expand the coverage of the sensorimotor cortex.

Functional connectivity was estimated using phase-locking value (PLV) in α and β bands, and network-based statistics identified subnetworks associated with motor tasks. Classification with sparse multinomial logistic regression (SMLR) and SVM achieved 75% accuracy in distinguishing left versus right foot motor imagery.

Beyond functional connectivity and classification models, cognitive engagement during motor imagery tasks represents a critical factor in BCI development for exoskeleton control. Despite its importance, publicly available EEG datasets in this context remain scarce. To address this, the EUROBENCH project in Spain [29] introduced a database designed to assess motor imagery during device control and gait attention on different surfaces. Validation of this dataset demonstrated classification accuracies above 70% for both motor imagery and gait attention tasks, making it a valuable resource for advancing EEG-based BCIs.

Finally, researchers at Yanshan University School of Electrical Engineering [30] applied the Disperse Common Spatial Pattern (DCSP) algorithm to optimize EEG channel selection, identifying those channels most relevant for motor imagery decoding. To overcome singularity issues, a regularized discriminant analysis was introduced, enhancing classification performance.

### 3.5. Artificial Intelligence-Based Signal Processing

A clear distinction must be made between conventional signal processing methods and those based on artificial intelligence, as both coexist in the literature and serve different methodological purposes. Conventional approaches typically involve manual or semi-automatic steps such as filtering, artifact rejection, and feature extraction using mathematical transforms (e.g., Fourier or wavelet), followed by classification with traditional models like Linear Discriminant Analysis (LDA) or Support Vector Machines (SVMs).

In contrast, AI-based processing refers to machine learning and deep learning techniques that automate feature learning directly from raw or minimally processed data. These include models such as convolutional neural networks (CNNs), long short-term memory networks (LSTMs), and hybrid architectures, which capture complex spatial, spectral, and temporal dependencies in EEG signals. This section focuses on the latter group, reviewing studies that leverage artificial intelligence to enhance decoding accuracy, generalizability, and real-time applicability in motor imagery classification.

Signal processing methods based on artificial intelligence have become essential for extracting meaningful features from EEG signals during motor imagery tasks. This subsection reviews key approaches that leverage machine and deep learning algorithms to enhance the interpretation of neural activity, covering both classical and advanced techniques applied in the time, frequency, and spatial domains. Special attention is given to widely adopted algorithms such as CSP, Filter Bank-CSP, and decomposition-based methods, which are fundamental for increasing the discriminability of motor-related brain patterns and optimizing BCI system performance [31].

**Figure 5:**
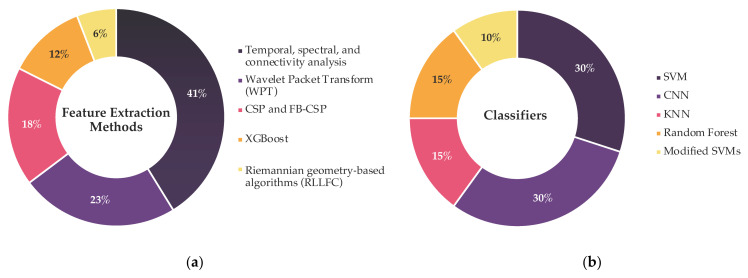
Summary of the main signal processing techniques applied in EEG-based BCIs for MI tasks. Part (**a**) shows feature extraction methods including both classical and AI-based approaches, while part (**b**) presents classification techniques based on AI models.

To provide a quantitative perspective, Figure 5 summarizes the application frequency of these techniques across the analyzed studies, highlighting the dominant methodologies for motor imagery feature extraction and offering insight into current trends in the field.

In Figure 5a, feature extraction techniques show a marked preference for time–frequency representations (e.g., wavelet transforms, WPD), decomposition methods (e.g., EMD/EEMD), and spatial filters (notably CSP and its variants), underscoring their utility in isolating motor-relevant EEG components.

Regarding classifier methods, Figure 5b highlights the predominance of SVMs and CNNs, reflecting a dual trend: SVMs remain popular for their robustness with small datasets and engineered features, while CNNs learn spatial–spectral representations directly from raw or minimally preprocessed signals. The high frequency of hybrid models also points to growing interest in temporal dynamics and cross-modal integration.

Advancing toward machine and deep learning algorithms for data processing, the classification of EEG signals obtained from lower-limb motor imagery experiments is commonly performed using techniques such as SVMs. In this regard, the study presented in [26] reports an accuracy of 88.43%, surpassing traditional signal-processing approaches. Following this same line, the research in [27] achieves a maximum accuracy of 91.66% using SVM and 90.33% with KNN.

On the other hand, the model proposed in [32], which also employs SVM, introduces a novel rehabilitation approach based on motor imagery for post-stroke hemiplegic patients, using EEG to decode normal gait and hemiplegic gait imagery. In this study, thirty subjects performed lower-limb coordination MI tasks while their EEG was recorded, with analyses conducted in the time domain, spectral domain, and brain connectivity.

The features extracted from the brain network, together with an SVM, enabled the classification of both gait types with an average accuracy of 92.96%. The results show that the θ and α bands, along with right frontal brain connections, are key for differentiating the analyzed motor imagery tasks.

Motor imagery (MI)-based brain–computer interfaces (BCIs) have gained momentum, but their deployment in real-world settings remains limited by the reliance on labeled data. Manual labeling of brain signals is labor-intensive, induces mental fatigue, and slows the development of accurate models [56]. To address this challenge, various approaches have been proposed.

Notably, ref. [33] proposed an online semi-supervised learning framework based on a regularized and weighted online sequential extreme learning machine (RWOS-ELM) as the core classifier. This model incrementally updates its parameters using balanced data chunks. In the initial phase, synthetic oversampling of the minority class is combined with a modified KNN, enabling effective EEG data augmentation and the construction of initial classifiers with improved segmentation and early motor imagery recognition [33].

EEG signal monitoring remains challenging due to high noise levels and the non-stationary nature of the signals. To address these limitations, ref. [34] proposed an automatic motor imagery classification method combining wavelet packet transform, statistical feature extraction, and a multilayer perceptron classifier. The most informative features were selected using the Kruskal–Wallis test and used to train and validate the neural network.

In a related study, Gu, et al. [28] analyzed cortical activity differences during motor imagery of left and right foot movements. EEG data from 64 channels in 11 subjects were used to construct a cortical source model covering 62 regions of interest. Functional connectivity analysis in the α and β bands identified relevant subnetworks, and classification using an SVM optimized with sparse multinomial logistic regression (SMLR-SVM) achieved 75% accuracy, providing valuable insights into the neuromotor mechanisms underlying lower-limb motor imagery.

As research progresses, the evaluation of neural network models for interpreting mental activity has become increasingly systematic. In this regard, ref. [35] compared 16 ANN architectures across 4 BCI paradigms, assessing their information representation capacity. Based on these findings, the authors proposed EEGNex, an optimized CNN that improved classification accuracy by 2.1% to 8.5% across different contexts, outperforming other evaluated models.

The study in [30] improved motor imagery classification by applying the Common Spatial Pattern (CSP) algorithm combined with sparse techniques and iterative search strategies. The classification stage used a regularized version of Linear Discriminant Analysis (LDA) to address singularity and enhance robustness. Evaluated on the BCI Competition IV dataset and a custom dataset, the method achieved a 10.75% increase in classification accuracy over the conventional CSP + LDA approach, demonstrating strong performance in both cases.

In the field of MI-based BCIs, deep learning (DL) decoders have demonstrated high performance. However, their deployment on portable devices remains limited due to high computational demands. The integration of these models into standalone microcontroller units (MCUs) has been scarcely explored. Addressing this gap, ref. [36] developed an EEG decoder based on a CNN with a spatial attention mechanism, optimized for operation on a fully integrated microcontroller (MCU). Trained on the GigaDB dataset (52 subjects), the model achieved an average accuracy of 96.75% using eight EEG channels, surpassing the EEG-Inception model, which reached 76.96% with six channels.

This work represents the first portable deep learning decoder for MI signals, demonstrating strong potential for AI-driven portable BCI devices. This study introduces the first portable deep learning decoder for MI signals optimized for real-time inference on MCUs. While training remains complex, the model’s compact design supports efficient deployment. Although transfer learning is not directly evaluated, strong cross-subject accuracy indicates the potential for future generalization [36].

Motor imagery-based research has also advanced in motor rehabilitation, particularly in supporting lower limb function. Some BCIs enable precise detection of movement intention, facilitating their application in clinical settings. Recent developments, such as those described in [37], have adopted multimodal strategies combining visual, auditory, functional electrical, and proprioceptive stimulation to optimize rehabilitation training.

Additionally, the Riemannian Local Linear Feature Construction (RLLFC) algorithm was applied to enhance signal decoding through unsupervised learning and weighted feature representation. Experimental validation demonstrated improved feature extraction and motor pattern classification compared with conventional methods, supporting the effectiveness of these approaches in clinical rehabilitation [37].

Additional contributions are exemplified by [31,38], who proposed combining temporal, spatial, and frequency domain features to improve EEG-based BCI performance. These studies highlight the importance of effective feature processing and selection in motor imagery classification. In particular, ref. [38] employed an optimized Extreme Gradient Boosting (XGBoost) algorithm for feature selection that reduced dimensionality and enhanced computational efficiency without sacrificing accuracy.

For classification, the RF algorithm was applied, recognized for its ability to handle large feature sets and minimize overfitting [38]. The study compared this approach with traditional feature selection methods commonly used in EEG processing, including ANOVA, forward sequential selection, recursive feature elimination (RFE), and LASSO. Experimental results showed that the combination of XGBoost and RF outperformed these classical methods, achieving accuracies of up to 94.44% and 88.72% on datasets IIIa and IVa of BCI Competition III, respectively [38].

The sharp rise of deep-learning classifiers (CNNs, LSTMs, ViT) from <15% of studies in 2020 to >45% in 2024 reflects three converging factors: (i) Commodity GPUs and mature libraries lowered entry barriers. (ii) DL models capture joint spatio-spectro-temporal patterns without extensive feature engineering. (iii) Recent compact architectures (e.g., EEG-Inception, EEGNeX, Prob-Sparse-CNN) achieve >90% accuracy with 6 to 8 channels and fit on microcontrollers, enabling real-time portable BCIs.

### 3.6. Motion Imagery (MI) Rehabilitation Applications in Lower Limbs

This subsection examines methods using motor imagery (MI) detection as an assistive mechanism in lower-limb neuromotor rehabilitation. These methods typically combine EEG-based BCI systems with various feedback technologies, such as robotic exoskeletons, to support or enhance motor recovery. The reviewed studies emphasize therapeutic strategies that enable active patient participation, where the user’s motor intent, detected via EEG signals, is translated into meaningful actions through external actuators or virtual environments. Such approaches aim not only to assist movement but also to stimulate neuroplastic changes and promote long-term functional improvements in gait and lower-limb coordination.

**Figure 6:**
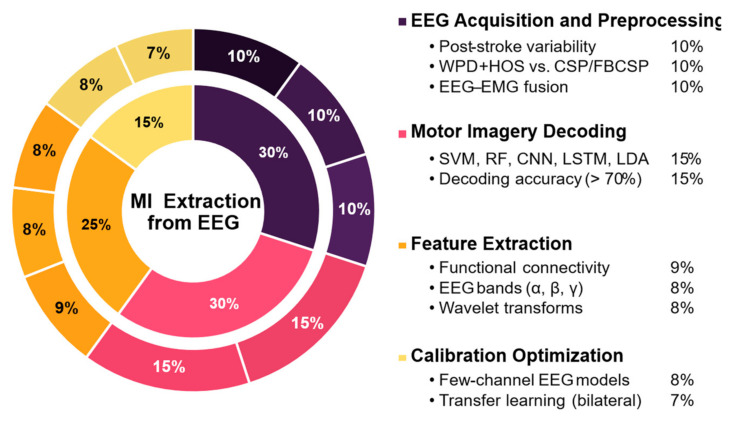
Overview of EEG signal processing methods used in lower-limb motor imagery rehabilitation. The figure illustrates the main approaches applied for acquiring, processing, and decoding EEG signals in MI-based rehabilitation systems.

To provide a general overview of methodological trends in this field, Figure 6 summarizes the main strategies used for EEG acquisition, processing, and decoding in lower-limb motor imagery rehabilitation. It offers a visual snapshot of how studies distribute their efforts across key signal processing stages, supporting the development of effective BCI-based interventions.

Figure 6 summarizes the main methods used for processing and decoding EEG signals in lower-limb motor imagery rehabilitation. EEG acquisition and preprocessing represent 30% of the approaches, addressing post-stroke variability (10%), method comparisons (10%), and EEG–EMG fusion (10%). Feature extraction accounts for 25%, focusing on frequency bands (8%), functional connectivity (9%), and multiresolution techniques (8%). Classification and interpretation make up 30%, using algorithms like SVM, RF, CNN, LSTM, and LDA (15%) with accuracies ranging from 70% to 94.3% (15%). The remaining 15% target calibration optimization, including transfer learning (7%) and low-channel models (8%) for portable BCI applications.

**Figure 7:**
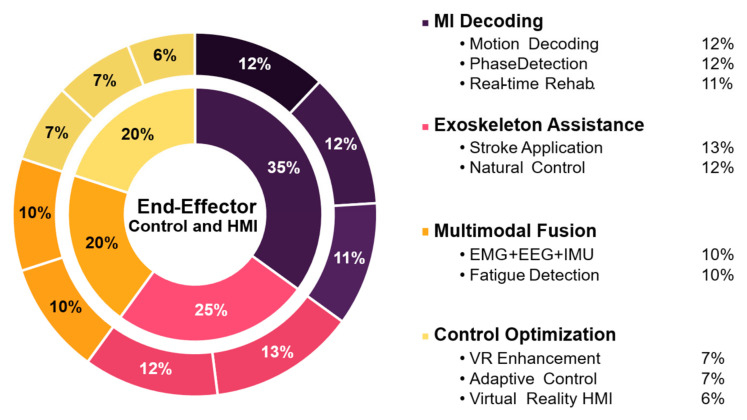
Summary of control strategies and human–machine interfaces in MI-driven lower-limb rehabilitation. The figure presents key approaches for decoding motor intention and implementing assistive feedback and control systems.

Building effective rehabilitation systems based on motor imagery requires not only accurate signal decoding, but also the integration of intuitive control strategies and responsive human–machine interfaces. Recent research has explored diverse approaches to translate motor intention into action, incorporating real-time feedback, multimodal inputs, and adaptive mechanisms. These strategies are summarized in Figure 7 to illustrate current directions in assistive system design for lower-limb rehabilitation.

Figure 7 illustrates the main strategies used for end-effector control and HMI implementation in lower-limb MI rehabilitation. These include movement intention decoding through EEG patterns and phase transitions; multimodal fusion of EEG, EMG, and biomechanical signals to enhance robustness; and the use of exoskeletons with intuitive, personalized control protocols. Additional approaches involve control optimization using virtual reality, neurophysiological markers like MRCP and ERD, and adaptive systems tailored to patient progress.

Brain–computer interfaces (BCIs) have emerged as innovative tools for communication and device control through brain signals, with applications in locomotion and mobility rehabilitation, particularly in addressing motor deficits caused by brain injuries. One of the main limitations of these systems is the prolonged calibration time required to train personalized machine learning models, which hinders their clinical adoption. To overcome this challenge, transfer learning methodologies have been explored as a strategy to reduce or eliminate the need for prior calibration sessions.

MI-based BCIs using EEG have shown strong potential in lower-limb exoskeleton rehabilitation by enabling direct decoding of motor intention. While early studies such as [40] inferred lower-limb intention from upper-limb EEG signals, more recent work demonstrates that decoding lower-limb motor imagery directly is more effective for control. In this context, ref. [39] proposed a CNN with sparse attention and spatiotemporal integration, achieving 89.04% offline accuracy, 57.28% real-time accuracy, and an information transfer rate of 7.70 bits/min, supporting its feasibility in rehabilitation scenarios.

Additionally, ref. [41] proposed a multimodal EEG–EMG fusion approach based on functional connectivity to improve early movement intention detection, particularly in transitions from sitting to standing. Connectivity networks within 1.5-s pre-movement windows were analyzed and classified using SVMs, achieving 94.33% accuracy with mutual information metrics. This method demonstrated robustness under fatigue and maintained high performance in spinal cord injury patients, confirming its potential for real-time rehabilitation applications.

A notable approach developed in [42] introduced a system that leverages EEG data recorded during bilateral movements assisted by an exoskeleton, where the movement of the healthy arm guides the affected arm. This strategy infers motor intentions without requiring extensive individual calibration. The classification model is first trained on bilateral movements and then applied to predict unilateral actions, achieving comparable performance to traditional models while using only 4 to 8 EEG channels, thus reducing the system’s complexity and enhancing usability.

In the context of lower-limb exoskeletons, ref. [43] proposed a multimodal human–machine interface (HMI) combining EEG and surface electromyography (sEMG) to improve gait intention detection. Data from 14 subjects were analyzed using both feature-based and end-to-end approaches. Signal fusion achieved a recognition rate of 89.5%, surpassing the performance of unimodal EEG or sEMG. Additionally, a temporal domain translation strategy improved cross-subject generalization, reaching 83.6% accuracy. These results confirm the practical viability of multimodal HMIs for functional rehabilitation and exoskeleton control using motor imagery.

Despite advances in neurorehabilitation, motor recovery in most patients remains incomplete. BCIs facilitate the translation of user intentions into real-time control commands, supporting the operation of exoskeletons, prostheses, orthoses, and wheelchairs, particularly in individuals with spinal cord injury or amputation [44]. Electroencephalography (EEG) has become a central tool in this context, enabling the development of motor imagery-based BCIs, which show great promise in the rehabilitation of stroke patients with motor impairments.

A key challenge in post-stroke rehabilitation is the variability and complexity of EEG signals due to cerebrovascular damage. Addressing this, ref. [45] evaluated various filtering and feature extraction techniques for classifying MI tasks. Wavelet packet decomposition (WPD) combined with Higher-Order Statistics (HOS) demonstrated superior performance, effectively capturing both temporal and nonlinear EEG features. Compared with CSP and Filter Bank-CSP, classification using RF showed that the WPD + HOS approach achieved the highest accuracy, exceeding 70%.

In this context, ref. [46] highlights that recognizing and classifying movements from EEG signals remains a key challenge in developing motor rehabilitation systems using BCIs. The study addressed this by classifying EEG signals into six hand movement types through machine learning and deep learning methods. The process involved EEG acquisition, preprocessing, and feature extraction across the α, β, θ, δ, and γ bands followed by model training. Deep learning models, including RNN-LSTM and CNN, were evaluated alongside traditional classifiers such as SVM and LDA. The approach achieved 70% accuracy for six hand movements in a single-subject scenario. For binary classifications, higher performance was observed, with CSP combined with RF and DWT with LSTM achieving 93% accuracy. Cross-subject classification was also explored, where CSP with LSTM reached 61% accuracy for four movement classes, indicating progress in improving model generalization [46].

Various MI-BCI systems have proven effective in controlling electromechanical devices for neuromotor rehabilitation. In this context, ref. [47] analyzed the role of EEG-based BCIs, particularly those using MI, highlighting their value as non-invasive and accessible tools for device control. The review examined over 220 studies on EEG signal processing in BCIs, covering key stages such as acquisition, preprocessing, feature extraction, and classification. Among the feature extraction methods, wavelet transform (WT) and wavelet packet transform (WPT) were noted for their effectiveness in analyzing non-stationary signals. In addition, the integration of artificial intelligence algorithms (spanning ML and DL models) has improved motor imagery classification.

Similarly, ref. [48] highlighted that while functional connectivity (FC) has been linked to upper-limb recovery after stroke, its role in lower-limb rehabilitation remains underexplored. EEG-based assessments of FC using electrodes over motor areas (C3, C4, FC3, FC4, FCz) have enabled correlations between neuronal activity and lower-limb function during recovery. Correlating amplitude envelopes across frequency bands has been key to developing predictive models, emphasizing the importance of intra- and interhemispheric connectivity in motor recovery.

In parallel, ref. [49] proposed a dynamic neural model simulating EEG signal during active lower-limb motor intention. Through experiments with 12 subjects, it was shown that virtual reality (VR) enhances EEG features such as movement-related cortical potentials and ERD, improving the early detection of movement intention. These results contribute to the development of more responsive and individualized rehabilitation systems.

Finally, ref. [50] reviewed advances in HMI recognition for improving human–robot interaction (HRI) in lower limb exoskeletons. Approaches were classified into musculoskeletal model-based and model-free methods, the latter including heuristics, machine learning, and deep learning. The review analyzed the use of EEG, EMG, and biomechanical signals as well as multimodal fusion and SVM classifiers, highlighting key challenges such as improving accuracy, system personalization, and real-world functional validation.

## 4. Discussion

This systematic review analyzed developments in EEG signal acquisition and intelligent processing for lower-limb motor imagery (MI) with a specific focus on their application in BCI systems for neurorehabilitation. The findings highlight three primary advances: (i) improved signal quality achieved through advanced filtering techniques, such as wavelet packet decomposition and empirical mode decomposition, along with optimized channel selection strategies based on functional relevance and iterative source localization; (ii) increasing adoption of neural network-based algorithms, particularly convolutional and recurrent architectures, which have enhanced classification accuracy; and (iii) growing implementation of multimodal and portable systems tailored for clinical settings.

### 4.1. Review Discussion and Perspectives

Advances in EEG signal acquisition and processing have strengthened the field of neuroengineering, particularly in motor imagery applications for BCIs [57]. A key trend identified in the reviewed literature is the integration of advanced filtering, channel selection, and signal decomposition techniques to address the inherent limitations of EEG, including low spatial resolution, non-stationarity, and low signal-to-noise ratio (SNR) [58]. Within this framework, studies such as Kardam [20] and Pawan [27] emphasize the role of decomposition algorithms and feature extraction methods in enhancing detection accuracy beyond conventional approaches.

In parallel, Mercado [21] and Blanco [22] demonstrate the effectiveness of neural networks, particularly LSTM architectures, in capturing complex brain patterns during dynamic motor tasks like walking and pedaling. A further relevant development is the adoption of multimodal strategies wherein the combination of EEG and EMG signals mitigates the limitations of each modality. As shown in [23,24], these fusion approaches enhance system robustness and accuracy, maintaining stable performance even under real-world conditions such as muscle fatigue [24].

Other studies have focused on developing clinically applicable solutions [38]. For example, the predictive model based on resting-state EEG proposed by [25] eliminates complex preprocessing, representing advances toward simplifying EEG use in hospital environments. However, critical challenges persist, including high inter- and intra-subject variability, the need for individualized calibration, and the limited availability of open-access databases reflecting realistic motor tasks [24]. In this regard, the EUROBENCH project [29] provides a valuable contribution, offering datasets that better capture real-world conditions.

Overall, the field is progressing toward more accurate, adaptive, and clinically viable BCI systems. Nevertheless, its consolidation requires the development of robust methodologies, comprehensive datasets, and standardized validation protocols to ensure generalizability and reliability [40]. Motor imagery remains a cornerstone of non-invasive neurorehabilitation strategies, and its continued exploration from diverse methodological perspectives holds great promise for advancing brain–technology interaction [17].

The application of artificial intelligence in processing EEG signals related to MI has advanced the development of BCIs, particularly for lower limb rehabilitation [37]. As shown in the reviewed studies, classification algorithms such as SVM, KNN, and deep neural networks have substantially outperformed traditional methods, achieving classification accuracies of up to 96.75% [36].

A major focus of this progress has been the optimization of feature extraction and selection by integrating temporal, spectral, and spatial information [30]. Techniques such as sparse CSP with regularization, the extraction of statistical indices from wavelet-transformed signals, and the use of XGBoost for dimensionality reduction have been essential for enhancing classifier performance [30]. Beyond improving accuracy, these methods have contributed to a deeper understanding of motor imagery-related brain activity, as evidenced by the identification of functional networks in the θ, α, and β bands.

Despite ongoing progress, manual labeling of EEG data remains a major bottleneck in the development of brain–computer interfaces. This process is time-consuming, labor-intensive, and prone to human error, which limits scalability and delays the deployment of adaptive, real-time systems. Semi-supervised strategies, such as the RWOS-ELM scheme [33], have partially addressed this issue by enabling real-time classifier updates with minimal labeled data, contributing to the development of adaptive and autonomous systems suitable for clinical use. The need for labeled data also imposes a high cognitive load on users during data collection, especially in clinical settings where patient fatigue must be minimized.

The implementation of deep learning models on portable devices has also been limited by computational constraints. Some studies, such as [36,39,46,47,50] have demonstrated the feasibility of integrating CNNs with attention mechanisms into microcontrollers (MCUs) and other computational devices, representing a major step toward the development of portable and accessible BCIs, particularly for individuals with reduced mobility.

Additionally, the integration of multimodal stimuli [23,24,44] with unsupervised learning algorithms highlights the potential of BCIs as intensive and personalized rehabilitation tools. These approaches, by combining sensory stimulation with accurate brain signal decoding, promote neuroplasticity and accelerate functional recovery in stroke and hemiplegia patients. Despite these advances, several challenges persist, including interindividual variability, the scarcity of standardized datasets, and the need for greater transparency in system operation [17].

### 4.2. Implications for Future Research and Clinical Practice

To advance the field toward practical applications, several directions should be prioritized. Future research should emphasize the development of interpretable models trained on large-scale, functionally rich databases and capable of adapting to diverse user profiles without compromising performance [59]. At the same time, greater efforts are needed to consolidate and promote the use of existing benchmark datasets, such as the BCI Competition series and EUROBENCH [29], by encouraging their adoption in cross-study evaluations and establishing them as reference standards for algorithm validation.

Moreover, the creation of new, publicly available datasets focused explicitly on lower-limb motor imagery under valid conditions would help bridge the gap between laboratory research and clinical deployment. This requires coordinated community initiatives to define standardized EEG acquisition protocols, task paradigms, and performance metrics that support robust comparison across systems and enhance reproducibility in neurorehabilitation research.

Validating systems across diverse populations with larger sample sizes and longitudinal follow-up is crucial, as is the exploration of lightweight, calibration-free BCI models suitable for real-world deployment in rehabilitation centers or home environments. Combining multimodal stimuli (visual, proprioceptive, and electrical) with unsupervised learning can boost functional recovery. Additionally, emerging strategies such as transfer learning, semi-supervised training, and task-specific model adaptation can lower barriers to clinical adoption and enable personalized therapy.

However, EEG-based BCIs have demonstrated potential in motor rehabilitation, particularly for patients with stroke or spinal cord injury [24,45,48]. A key challenge identified in the literature is the reduction in calibration time for personalized models, which continues to limit clinical adoption [49]. Transfer learning strategies have emerged as a promising solution, enabling the reuse of models trained on bilateral tasks to predict unilateral motor intentions without additional calibration. This approach improves clinical efficiency and enhances the user experience, increasing the practical viability of BCI systems [42,52].

From a methodological standpoint, the reviewed studies reflect progress in EEG signal processing. The integration of wavelet packet decomposition [27,34,47] and Higher-Order Statistics (HOS) [27] has improved the extraction of temporal and nonlinear features, surpassing conventional approaches such as CSP and FBCSP [30,46]. Additionally, the application of robust classifiers like RF and deep learning models using CNNs and LSTMs [36,39,46,47,50] has enhanced the classification of multiple motor imagery tasks, including in cross-subject scenarios where generalization is critical.

Despite these technical advances, motor recovery outcomes remain limited in many cases, highlighting the need for strategies that translate improved detection accuracy into functional gains [60]. Functional connectivity (FC) is proving to be a key metric in lower-limb rehabilitation, as increased intra- and interhemispheric FC has been linked to improved recovery outcomes [28,41,48]. These findings point to the potential for personalized interventions guided by neurophysiological biomarkers.

Finally, the field is advancing toward more efficient, adaptive, and clinically viable BCI systems. The combination of improved acquisition methods, advanced processing techniques, and robust learning algorithms is helping to overcome traditional limitations [61].

Nonetheless, critical challenges persist, including interindividual EEG variability and the limited use of existing large-scale resources. Although initiatives such as Mother of All BCI Benchmarks (MOABB) [62] and Braindecode [63] provide valuable multicenter datasets and standardized evaluation frameworks, their adoption across the motor imagery research community remains inconsistent, highlighting the need for broader integration and protocol harmonization [64].

### 4.3. Limitations of the Evidence Base and Review Process

Despite promising developments, the reviewed literature exhibits substantial heterogeneity in methodologies, including EEG acquisition protocols, classifier architectures, and performance metrics. Sample sizes were often small, and population characteristics (e.g., healthy vs. clinical subjects) were inconsistently reported. Few studies incorporated long-term follow-up or evaluated their systems in valid rehabilitation contexts.

Furthermore, the absence of standardized datasets and evaluation frameworks hampers cross-study comparisons and limits the generalizability of findings. Only a limited number of studies utilized open-access EEG datasets tailored for lower-limb motor imagery, underscoring the need for increased dataset transparency and broader community sharing [29].

In terms of the review process itself, no formal risk of bias assessment was conducted, and a quantitative synthesis or meta-analysis was not performed due to the methodological diversity of the included studies. While this review qualitatively identifies trends across the literature, it does not provide pooled effect sizes or standardized performance benchmarks. Additionally, the review was not prospectively registered in a systematic review database, and no formal protocol was prepared. Although the authors collaborated on study selection and data extraction, the absence of formal registration and protocol may affect reproducibility and increase the potential for selection bias.

The review covers only publications from 2020 to 2025. Therefore, observed patterns should be interpreted as a contemporary snapshot rather than a long-term trend.

## 5. Conclusions

This review has systematically analyzed advances driven by artificial intelligence methodologies in EEG signal acquisition, processing, and classification, particularly emphasizing lower-limb motor imagery for brain–computer interfaces (BCIs) and motor imagery (MI). Significant strides have been achieved by integrating advanced filtering techniques, optimal channel selection, and sophisticated signal decomposition methods, effectively overcoming traditional EEG limitations such as low spatial resolution, signal non-stationarity, and poor signal-to-noise ratios.

Machine learning algorithms, notably SVM, RF, CNN, and LSTM networks, have increased the accuracy and reliability of motor imagery classification tasks. These developments have consistently demonstrated classification accuracies exceeding 90%, with some studies reporting performances as high as 96.75%. Additionally, multimodal fusion strategies combining EEG with electromyography (EMG) and biomechanical signals have been shown to enhance system robustness and reliability, particularly under challenging conditions such as muscle fatigue and real-time rehabilitation tasks.

Statistical analysis from the reviewed literature indicates a balanced research focus across EEG acquisition and preprocessing methodologies (approximately 30%), feature extraction techniques such as wavelet packet decomposition and frequency band analysis (25%), and classification methods leveraging advanced machine learning and deep learning approaches (30%). Transfer learning and semi-supervised methods, in particular, have emerged as effective strategies to reduce calibration time, improving the clinical viability and user acceptance of BCI systems.

A key trend in the field is the shift toward lightweight, low-power BCI devices optimized for efficient operation with fewer EEG channels. This approach reduces costs and infrastructure requirements while enabling integration into portable, microcontroller-based systems, extending their use beyond laboratory environments.

In terms of broader societal and clinical impacts, these technological advancements provide concrete opportunities to enhance the quality of life for individuals with motor disabilities, facilitating intuitive and non-invasive interaction with their environment. Implementation in home environments, rehabilitation centers, and educational settings underscores the potential of BCIs in promoting social inclusion, personal autonomy, and personalized healthcare.

Despite the notable progress, several persistent challenges remain. High inter- and intra-subject variability, limited standardized datasets reflective of realistic motor tasks, heavy reliance on labeled data, and the need for improved model interpretability and adaptability constitute significant barriers to broader clinical translation. Addressing these challenges will require more than the availability of large-scale, multicentric datasets, standardized evaluation protocols, and transparent AI models. Initiatives such as MOABB and Braindecode have begun to provide these resources. However, a clearer understanding is also needed of why such tools remain underutilized in current studies. Placing greater emphasis on accessibility, comprehensive documentation, and alignment with clinically relevant tasks may be essential to promoting broader adoption and enabling the development of adaptive systems capable of real-time personalization.

Therefore, future work must validate these findings in multicenter clinical trials and develop standard, publicly available benchmark datasets and BCI models. Exploration of the dynamic personalization of interfaces, advanced multimodal signal integration, adaptive feedback mechanisms, and continuous learning systems represent promising pathways to bridging the gap between laboratory research and real-world clinical applications.

It is important to recognize that the integration of neuroscience with fields such as computer science and cognitive engineering provides a solid foundation for the development of human-centered, accessible, and inclusive technologies. Continued interdisciplinary advancements promise to refine brain–computer interfaces (BCIs), supporting more effective functional recovery, improved communication, and enhanced user autonomy.

Building on the insights of this review, future research should focus on the development of portable, multimodal BCI systems for lower-limb neurorehabilitation grounded in advances in computer science and cognitive engineering. These systems should integrate EEG and EMG signals, utilize machine or deep learning methods to reduce calibration time, and be optimized for real-time inference on low-power embedded platforms.

The development and refinement of AI-based models, including lightweight convolutional and recurrent architectures, attention mechanisms, and domain adaptation methods, will be essential to improving robustness, personalization, and cross-subject generalization. Validation should be conducted under valid conditions using functionally meaningful tasks in clinical populations, particularly individuals with stroke or spinal cord injury. This research direction directly addresses critical barriers to clinical translation and paves the way for accessible, adaptive, and intelligent BCI technologies that support effective neurorehabilitation in real-world settings.

## Figures and Tables

**Table 1:** Database-specific search strategies and query formulations.

Database	Search Query
Scopus	(TITLE-ABS-KEY ((“Electroencephalography” OR “EEG” OR “Brain”) AND (“signal” OR “Signal acquisition” OR “Data recording” OR “Processing” OR “Analysis”) AND (“Motor imagery” OR “Motor intention” OR “Movement imagination” OR “Mental rehearsal” OR “Motor tasks”) AND (“Artificial intelligence” OR “Machine learning” OR “Deep learning” OR “Pattern” OR “Classification” OR “Neural networks”) AND (“Brain-computer interface” OR “BCI” OR “Neural interface” OR “Brain-machine interface” OR “BMI” OR “Neuro*”) AND (“Lower limbs” OR “Legs” OR “Foot movements” OR “Gait analysis” OR “Walking” OR “lower”))) AND PUBYEAR > 2019 AND PUBYEAR < 2026 AND (LIMIT-TO (SUBJAREA, “COMP”) OR LIMIT-TO (SUBJAREA, “ENGI”)) AND (LIMIT-TO (DOCTYPE, “ar”) OR LIMIT-TO (DOCTYPE, “re”)) AND (LIMIT-TO (LANGUAGE, “English”))
IEEE Xplore	((“Electroencephalography” OR “EEG” OR “Brain”) AND (“Signal” OR “Signal acquisition” OR “Data recording” OR “Processing” OR “Analysis”) AND (“Motor imagery” OR “Motor intention” OR “Movement imagination” OR “Mental rehearsal” OR “Motor tasks”) AND (“Artificial intelligence” OR “Machine learning” OR “Deep learning” OR “Pattern recognition” OR “Classification” OR “Neural networks”) AND (“Brain-computer interface” OR “BCI” OR “Neural interface” OR “Brain-machine interface” OR “BMI” OR “Neuro*”) AND (“Lower limbs” OR “Legs” OR “Foot movements” OR “Gait analysis” OR “Walking” OR “Lower”)) Filters Applied: Conferences, Range 2020–2025

Note: The token “Brain” was included to retrieve papers that use the full phrase “brain–computer interface” yet omit the acronym BCI. Because the string was conjunctively combined with “EEG” OR “Electroencephalography”, records lacking an EEG component were excluded at the title/abstract stage.

**Table 2:** Overview of the main features of the studies selected for this review.

Study	Year	Study Design	PCPs	Strategy	Main Contribution or Milestone	Sample Size	Classes	Feature Types	Used Techniques	Data Type
[16]	2023	REV	NP	Publication trend analysis by country/year since 2019	Proposes a theoretical BCI architecture as a potential solution.	NA	NA	NA	NA	NA
[17]	2024	EXP + COMP	HS	Technical (non-medical) intervention	Average classification accuracy: 95% for MI.	15	2	NS	Multiple ML methods	RAW
[18]	2022	REV	NP	Document analysis of 22 relevant articles	Highlights the need for algorithm selection aligned with the goal and interpretability alongside accuracy.	NA	NA	NA	NA	NA
[19]	2022	REV	NP	Analytical and methodological intervention	Specific recommendations based on movement type/intensity. Compares methods’ pros and cons.	NA	NA	NA	NA	NA
[20]	2023	EXP + COMP	NP	EEMD + NN over existing EEG	Improved accuracy by 15% over EMD.	NS	2	EEMD	ANN (DL)	PPD
[21]	2021	EXP	HS	Multilayer perceptron (MLP) for torque decoding	More accurate for right leg.	NS	2	EEG	MLP (DL)	RAW
[22]	2024	EXP	HS	LSTM for lower-limb kinematics	Moderate accuracy for functional classification.	NS	2	EEG	LSTM (DL)	RAW
[23]	2020	EXP	HS	Multimodal and HMI exoskeleton control	Fusion improves accuracy and reliability.	NS	2	EEG + EMG	Multiple ML methods	RAW
[24]	2021	EXP	HS	Fusion via DCA classified by LDA	Improved accuracy from 89% to 97%.	28	2	EEG + EMG	DCA + LDA	PPD
[25]	2023	EXP + COMP	PS	Resting-state EEG (60s)	Accuracy: 87.50%.	NS	2	EEG signal time-series	LSTM (DL)	RAW
[26]	2023	EXP + COMP	HS	SVM optimized with particle swarm	Accuracy: 88.43%, improvement of 3.35–5.41%.	NS	2	TCS + PSO	SVM (ML)	PPD
[27]	2023	EXP + COMP	NP	WPD for features, SVM and KNN	Max acc: SVM 91.66%, KNN 90.33%.	NS	2	Wavelet features	SVM, KNN (ML)	PPD
[28]	2020	EXP + COMP	HS	PLV to create brain networks, SMLR + SVM	Accuracy up to 75%.	11	2	PLV (α and β bands)	SMLR + SVM (ML)	PPD
[29]	2023	EXP	HS	EEG recording during exoskeleton MI	Classification accuracy above 70%.	NS	2	EEG	Multiple ML methods	RAW
[30]	2020	EXP + COMP	NP	Sparse CSP + LDA	Accuracy improvement of 10.75%.	NS	2	CSP Features	LDA (ML)	PPD
[31]	2023	COMP	NP	Comparison of algorithms for BCI	Best accuracy: 89.7%.	NS	>2	Mixed features	Multiple ML methods	PPD
[32]	2021	EXP + COMP	PS	SVM trained with spatial/network features	Best accuracy: 92.96%.	30	2	CSP Features	SVM (ML)	PPD
[33]	2023	COMP	HS	RWOS-ELM + SMOTE + ENN online learning	Error reduction and stabilization.	6	>2	EEG signal time-series	RWOS-ELM (ML)	RAW
[34]	2022	EXP + COMP	HS	CWT for TF maps; ViT and ResNet	Best accuracy: 97.33%.	NS	4	CWT	ViT, ResNet (DL)	PPD
[35]	2023	COMP	NP	EEGNeX ConvNet vs. 16 DL models	Accuracy gains: 2.1–8.5%.	NS	11	ConvNet features	EEGNeX (DL)	PPD
[36]	2023	COMP	HS	CNN + spatial attention vs. EEG-Inception	Accuracy: 96.75%.	52	2	EEG maps	CNN (DL)	RAW
[37]	2023	COMP	HS	RLLFC + SVM	Accuracy: 88.4%.	20	2	Riemannian + Spatial	SVM (ML)	PPD
[38]	2022	COMP	NP	XGBO + Random Forest	Acc: 94.44% (IIIa), 88.72% (IVa).	NS	2	Mixed features	XGBoost + RF (ML)	PPD
[39]	2025	COMP	HS	CNN + Prob-Sparse Attention	Offline: >89%; online: 57.28%.	NS	2	Spatio-temporal	CNN + Attention (DL)	RAW
[40]	2020	REV	NP	Review of BCI system development trends	Guidelines for making BCI systems more accessible and collaborative.	NA	NA	NA	NA	NA
[41]	2025	EXP	HS + PS	EEG–EMG fusion, SVM voting	Fusion acc: 94.33% (HS), 87.54% (SCI).	13	3	EEG + EMG	SVM (ML)	PPD
[42]	2024	COMP	HS	Transfer learning for classifier	Best accuracy: 84.5%.	8	2	EEG	Transfer Learning (DL)	PPD
[43]	2024	EXP + COMP	HS	Cross-subject neural network pipeline	Identification rate: 83.6%.	14	2	NS	ANN (DL)	RAW
[44]	2021	REV	NP	Multimodal, multi-stage strategy with neuroimaging transitioning to mobile systems; protocol standardization	Proposes clinical adoption roadmap with multimodal methods and protocols.	NA	NA	NA	NA	NA
[45]	2022	EXP	PS	RF with WPD + HOS	Accuracy > 70%.	6	2	WPD + HOS	Random Forest (ML)	PPD
[46]	2023	EXP + COMP	HS	Multiple classifiers	Acc. Binary: 93%; Multiclass: 70%; Cross-subject: 61%.	1	4	CSP, DWT, TF	ML/DL mixed	PPD
[47]	2023	REV	NP	Feature extraction and classifiers benchmarking	ML performs well with few channels; DL improves accuracy but needs more data/computation time.	NA	NA	NA	WT, WPT, SVM, LDA, RF, KNN, CNN	NA
[48]	2021	EXP	PS	Multiple regression on FC	Early FC predicts LL recovery.	24	NS	NS	Linear regression	RAW
[49]	2023	EXP + COMP	HS	MRCP and ERD in VR	VR improved peak amplitude, SNR.	12	2	MRCP, ERD	ANN (DL)	RAW
[50]	2023	REV	NP	Multisensor: EEG, EMG. Biomechanics.	Highlights preprocessing and benchmarking needs.	NA	NA	NA	SVM, RF, KNN, LD (ML), ANN (DL)	NA

## Data Availability

This review is based entirely on previously published studies. No new data, code, or software were generated. A list of included and excluded studies, along with the full data extraction form, is available from the corresponding author upon reasonable request.

## Abbreviations

The following abbreviations are used in this manuscript:

AI	Artificial intelligence
(A)NN	(Artificial) neural network
ANOVA	Analysis of Variance
BCI	Brain–computer interface
CNN	Convolutional neural network
(D)CSP	(Disperse) Common Spatial Pattern
CTR	Continuous-trajectory reconstruction
DCA	Discriminant Correlation Analysis
DL	Deep learning
DSP	Digital Signal Processor
DWT	Discrete Wavelet Transform
EEG	Electroencephalography
EEG-BCI	Electroencephalogram-based brain–computer interface
(E)EMD	(Ensemble) empirical mode decomposition
EMG	Electromyography
ERD	Event-related desynchronization
ERS	Event-related synchronization
FBCSP	Filter Bank Common Spatial Pattern
FC	Functional connectivity
FFT	Fast Fourier Transform
FMRI	Functional Magnetic Resonance Imaging
FNIRS	Functional Near-Infrared Spectroscopy
HMI	Human–machine interface
HOS	Higher-Order Statistics
HRI	Human–robot interaction
IEEE	Institute of Electrical and Electronics Engineers
KNN	K-Nearest Neighbors
LDA	Linear Discriminant Analysis
LSTM	Long short-term memory
MCU	Microcontroller unit
MDF	Multidomain feature
MEG	Magnetoencephalography
MI	Motor imagery
MI-BCI	Motor imagery brain–computer interface
ML	Machine learning
MLP	Multilayer perceptron
MOABB	Mother of All BCI Benchmarks
MRCP	Magnetic Resonance Cholangiopancreatography
OCS	Optimal channel set
PCC	Pearson’s correlation coefficient
PLV	Phase-locking value
PRISMA	Preferred Reporting Items for Systematic Reviews and Meta-Analyses
PSO	Particle swarm optimization
RF	Random Forest
RFE	Recursive feature elimination
RLLFC	Riemannian Local Linear Feature Construction
RNN	Recurrent neural network
RWOS-ELM	Regularized weighted online sequential extreme learning machine
SMLR	Sparse multinomial logistic regression
SMLR-SVM	Sparse multinomial logistic regression Support Vector Machine
SNR	Signal-to-noise ratio
SVM	Support Vector Machine
TBI	Traumatic brain injury
TCS	Traditional channel set
ViT	Vision Transformer
VR	Virtual reality
WPD	Wavelet packet decomposition
WPT	Wavelet packet transform
WT	Wavelet transform
